# Development of a stacked ensemble model for risk stratification of chronic GVHD after allogeneic HSCT: Japanese nation-wide cohort study

**DOI:** 10.3389/fimmu.2026.1883360

**Published:** 2026-08-25

**Authors:** Makoto Iwasaki, Junya Kanda, Fumihiko Kimura, Sachiko Seo, Yoshiko Atsuta, Naoyuki Uchida, Noriko Doki, Takahiro Fukuda, Tetsuya Nishida, Yuta Katayama, Masatsugu Tanaka, Taro Tochigi, Satoshi Yoshihara, Yoshinobu Kanda, Masashi Sawa, Makoto Onizuka, Moeko Hino, Koji Kawamura, Ken Tabuchi, Akifumi Takaori-Kondo

**Affiliations:** 1 Department of Hematology and Oncology, Graduate School of Medicine, Kyoto University, Kyoto, Japan; 2 Division of Hematology, Department of Internal Medicine, National Defense Medical College, Tokorozawa, Japan; 3 Department of Hematology and Oncology, Dokkyo Medical University School of Medicine, Tochigi, Japan; 4 Japanese Data Center for Hematopoietic Cell Transplantation, Nagakute, Japan; 5 Department of Registry Science for Transplant and Cellular Therapy, Aichi Medical University School of Medicine, Nagakute, Japan; 6 Department of Hematology, Federation of National Public Service Personnel Mutual Aid Associations TORANOMON HOSPITAL, Tokyo, Japan; 7 Hematology Division, Tokyo Metropolitan Cancer and Infectious Diseases Center, Komagome Hospital, Tokyo, Japan; 8 Department of Hematopoietic Stem Cell Transplantation, National Cancer Center Hospital, Tokyo, Japan; 9 Department of Hematology, Japanese Red Cross Aichi Medical Center Nagoya Daiichi Hospital, Nagoya, Japan; 10 Department of Hematology, Hiroshima Red Cross Hospital & Atomic-bomb Survivors Hospital, Hiroshima, Japan; 11 Department of Hematology, Kanagawa Cancer Center, Yokohama, Japan; 12 Department of Hematology, Hamanomachi Hospital, Fukuoka, Japan; 13 Department of Hematology, Hyogo Medical University Hospital, Nishinomiya, Japan; 14 Division of Hematology, Jichi Medical University Saitama Medical Center, Saitama, Japan; 15 Department of Hematology and Oncology, Anjo Kosei Hospital, Anjo, Japan; 16 Department of Hematology/Oncology, Tokai University School of Medicine, Isehara, Japan; 17 Department of Pediatrics, School of Medicine, Chiba University, Chiba, Japan; 18 Division of Hematology and Clinical Laboratory Medicine, Tottori University, Yonago, Japan

**Keywords:** acute graft-versus-host disease, allogeneic hematopoietic stem cell transplantation, chronic graft-versus-host disease, machine learning, risk stratification, stacked ensemble model

## Abstract

Effective risk stratification is vital for donor selection and treatment strategies in allogeneic hematopoietic stem cell transplantation (HSCT). We developed a prediction model for mid- to long-term outcomes after HSCT using a stacked ensemble model (SEM). Using data from the Japanese Transplant Registry Unified Management Program, we analyzed 14,430 patients alive without chronic GVHD (cGVHD) or relapse on day 100 after their first HSCT for hematologic malignancies between 2010 and 2018, predicting 24-month outcomes from pretransplant variables and posttransplant acute GVHD (aGVHD) information, including its treatment, accrued by days 30, 60, and 100. Data were randomly divided into training (80%) and validation (20%) sets, with 14 pretransplant risk factors as input variables. SEM achieved the highest C-index across evaluated endpoints, cGVHD, non-relapse mortality (NRM), and all-cause mortality (ACM), significantly exceeding the weaker learners for all endpoints and, using pretransplant factors, the strongest learner for NRM and ACM as well (both p=0.01), with a smaller, non-significant margin for cGVHD (C-index for cGVHD/NRM/ACM—SEM: 0.574/0.652/0.642, Cox-PH: 0.553/0.635/0.615, Random Survival Forest: 0.564/0.638/0.613, XGBoost: 0.556/0.633/0.627, Dynamic-DeepHit: 0.508/0.607/0.564). The C-index increased as posttransplant aGVHD information accrued (day 30: 0.581/0.655/0.649; day 60: 0.602/0.688/0.656; day 100: 0.606/0.696/0.664). Grade III to IV aGVHD showed predictive contribution to NRM, ultimately impacting ACM. Our SEM-based model offers a useful framework for predicting post-HSCT outcomes and highlights the critical impact of early aGVHD events on long-term prognosis.

## Highlights

- The SEM matched or significantly exceeded every individual learner, including the cause-specific Cox proportional hazards model and advanced machine-learning survival models.
- SHAP from SEM showed grade III–IV aGVHD impacts NRM/ACM, whereas grade II–IV aGVHD and treatment response affect cGVHD risk.

## Introduction

Effective risk stratification is essential for selecting suitable donors and formulating treatment strategies in allogeneic hematopoietic stem cell transplantation (allo-HSCT). Several pretransplant risk models have been developed to predict posttransplant outcomes based on clinical factors available before transplantation (1, 2). However, accurately forecasting mid- to long-term prognosis remains a significant challenge.

Chronic graft-versus-host disease (cGVHD) is a major complication that arises beyond the early posttransplant phase, following successful engraftment and resolution of acute GVHD (aGVHD), and is associated with poor long-term outcomes (3). Although cGVHD shares many risk factors with aGVHD, its development is also influenced by the early posttransplant clinical course—particularly the occurrence and severity of aGVHD. Immunological changes during this period play a critical role in the pathogenesis of cGVHD, and numerous biomarkers have been proposed and investigated in previous studies. While it is well recognized that posttransplant events influence cGVHD development, the extent to which these events can improve prediction of cGVHD and long-term outcomes remains under investigation.

Mechanistically, cGVHD reflects a sustained alloimmune response orchestrated by aberrant T- and B-cell reconstitution after engraftment (4, 5). CD4+ T-helper cells skew toward Th1 and Th17 phenotypes, whereas regulatory T-cell recovery lags, tilting the effector–regulator balance toward chronic tissue injury (6). Dysregulated B-cell reconstitution, with elevated B-cell activating factor (BAFF), persistence of donor B-cell clones, and emergence of autoreactive antibodies, sustains the alloimmune drive (7). These cellular changes are accompanied by a soluble inflammatory signature, including the IFN-γ-inducible chemokine CXCL10, that has been associated with subsequent cGVHD risk when measured around 3 months posttransplant, particularly in combination with reduced plasmacytoid dendritic cell counts (8). Together, these features describe a multidimensional immunological state that no single biomarker can fully capture. Machine-learning approaches that integrate clinical events with such readouts therefore offer a natural framework for risk stratification: rather than searching for a single causal predictor, an ensemble can weight many partially predictive signals and recover the joint structure that links early posttransplant immune perturbation to later cGVHD onset. The present analysis takes a first step toward that integration by quantifying how much predictive accuracy is gained when posttransplant aGVHD events, themselves proxies for early immune dysregulation, are added to pretransplant variables.

To address this, we apply a stacked ensemble model (SEM) capable of handling right-censored data and accounting for competing risks, which we previously established and validated for GVHD-free, relapse-free survival (GRFS) prediction using both classical statistical and machine learning approaches (9). The ensemble integrates methods spanning from classical, population-based parametric models to modern, individualized, and non-parametric approaches, optimizing predictive performance by leveraging the strengths of each. Here, we apply this framework to three mid-to-long-term outcomes—chronic GVHD (primary endpoint), non-relapse mortality (NRM), and all-cause mortality (ACM)—and quantify how much predictive accuracy is gained by incorporating early posttransplant acute-GVHD events.

## Methods

### Population

**Figure 1:**
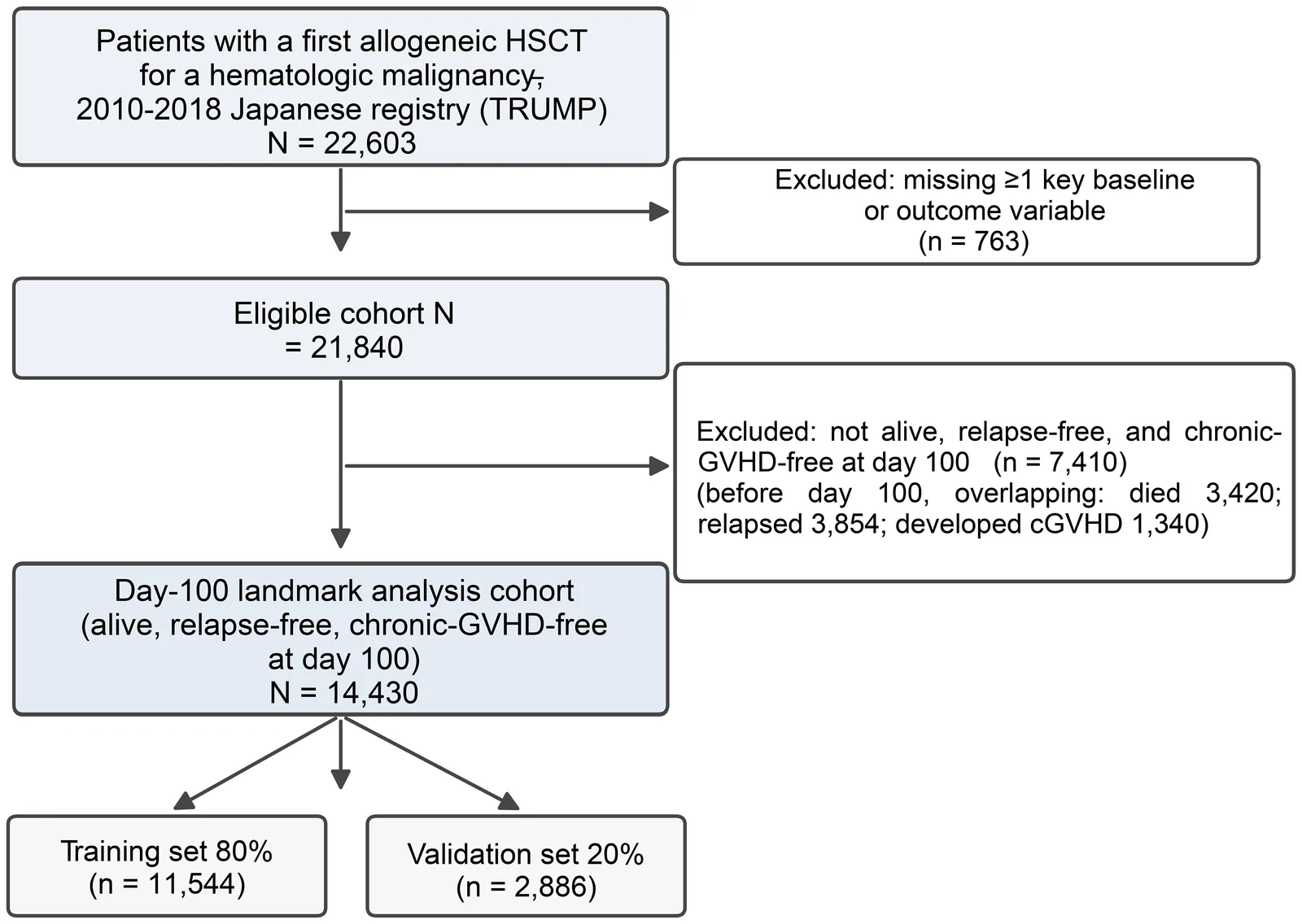
Patient-selection flow diagram.

All transplantation data in Japan are annually collected at the Japanese Data Center for Hematopoietic Cell Transplantation (JDCHCT). Using Japanese registry data, the Japanese Transplant Registry Unified Management Program, we identified 22,603 patients who underwent a first allo-HSCT for a hematologic malignancy between 2010 and 2018. After excluding 763 patients with a missing key baseline or outcome variable (eligible cohort, 21,840), we applied a day-100 landmark design (10, 11), excluding a further 7,410 patients who were not alive, relapse-free, and chronic-GVHD-free at day 100 (before day 100, overlapping: 3,420 died, 3,854 relapsed, and 1,340 developed chronic GVHD), yielding the day-100 landmark cohort of 14,430 patients (training set, n=11,544; validation set, n=2,886). The study was conducted according to the Declaration of Helsinki and was approved by the institutional review boards at Kyoto University Hospital and all other participating centers. Patient selection is summarized in **Figure 1**.

### Endpoint and definitions

Primary endpoint was cGVHD and secondary endpoints were NRM and ACM. Chronic GVHD was diagnosed by the attending physicians at each transplant center in accordance with the National Institutes of Health (NIH) consensus criteria and reported to the registry; the endpoint modeled here was the occurrence of any chronic GVHD. Each endpoint was measured from transplantation: chronic GVHD (primary endpoint) is the time to clinically diagnosed chronic GVHD, with death without chronic GVHD as the competing event; non-relapse mortality (NRM) is the time to death without prior relapse, with relapse as the competing event; and all-cause mortality (ACM) is the time to death from any cause (equivalently, 1 − overall survival), with the survival function estimated by the Kaplan–Meier method. Predictions used pretransplant covariates and, sequentially, acute-GVHD information accrued by days 30, 60, and 100. Data were randomly split into the training set (80% of the dataset) and the validation set (20%).

### Stacked ensemble model

A stacked ensemble of multiple machine-learning algorithms for right-censored data was applied to develop a model. Formally, an ensemble model is a model that combines the predictions from multiple trained models. A stacked ensemble model is a variation of the ensemble method and uses an algorithm that takes the outputs of submodels as inputs and learns the optimal way to combine the input predictions (12). Predictions are first generated using different algorithms, including cause-specific Cox Proportional Hazard (Cox-PH), Random Survival Forest, Dynamic-DeepHit, ADABoost, XGBoost, Extra Tree Classifier, Bagging Classifier, and Gradient Boosting Classifier (13–19). A meta-model is subsequently trained, using these predictions as inputs, to generate the final prediction. Each base learner was configured for the competing-risks structure of the data: Cox PH and Random Survival Forest were specified with cause-specific hazards for cGVHD versus death without cGVHD; Dynamic-DeepHit jointly modeled cause-specific cumulative-incidence functions; and the tree-based classifiers (AdaBoost, XGBoost, Extra Trees, Bagging, and Gradient Boosting) were trained on discretized time intervals with cause-specific class labels (20). Within the training set, base-learner hyperparameters were chosen by fivefold cross-validation on a grid limited to a small number of clinically meaningful values (tree depth, learning rate, number of estimators, and dropout for the neural network) to minimize overfitting on this single-cohort dataset. The meta-learner was a non-negative weighted average of the base-learner predictions: within the training set, out-of-fold predictions were generated by five-fold cross-validation; the five best-performing base learners, ranked by their out-of-fold concordance index, were retained; and their weights were estimated by derivative-free Nelder–Mead optimization to maximize the out-of-fold concordance index, subject to a non-negativity constraint and normalization to sum to one. The frozen ensemble was then applied to the held-out validation set. We chose this stacked configuration in preference to a simple unweighted average because the latter is degraded by weak or correlated base learners, whereas the stacking objective penalizes them automatically.

Data were randomly split into the training set (80% of the dataset) and the validation set (20%). We treated patient age and time from diagnosis to transplant as continuous variables and donor/graft source, performance status, ABO type matching, sex matching, disease/status groups based on refined Disease Risk Index, Hematopoietic Cell Transplantation-specific Comorbidity Index, usage of total body irradiation, conditioning intensity, T-cell depletion *in vivo*, human leukocyte antigen (HLA) locus matching, the number of HLA mismatching, and CMV serostatus as categorical variables. To evaluate predictive accuracy at days 30, 60, and 100 posttransplant, we considered acute graft-versus-host disease (aGVHD) and its treatment, including grade II to IV aGVHD occurrence, grade III to IV aGVHD occurrence, stage of aGVHD before and after treatment, corticosteroid treatment, anti-thymocyte globulin treatment, and treatment response of first-line therapy for aGVHD. Missing covariate values were handled by single imputation, with continuous variables replaced by the training-set median and categorical variables by the training-set mode; imputation parameters were estimated on the training data only and applied unchanged to the validation set.

### Other statistical considerations

Descriptive statistics were used to summarize patient characteristics. Comparisons of intergroup distributions were performed with the chi-square test or Fisher’s exact test for categorical variables and the Kruskal–Wallis test for continuous variables. Overall survival, the complement of all-cause mortality, was estimated using the Kaplan–Meier method. Cumulative incidences for relapse, NRM, and GVHD were calculated using the cumulative incidence function to account for competing risks. Competing events were death without relapse for relapse, relapse for NRM, and death without GVHD for chronic GVHD. P values <.05 were considered significant. All statistical analyses were performed with Python version 3.7 (Python Software Foundation, Fredricksburg, VA). All models were implemented in Python using the following publicly available open-source libraries: the cause-specific Cox proportional-hazards model and Random Survival Forest with scikit-survival, Dynamic-DeepHit with pycox, and the AdaBoost, Extra Trees, Bagging, and Gradient Boosting classifiers with scikit-learn and the xgboost library; meta-learner weights were optimized with SciPy and SHAP values with the shap package, using a fixed random seed throughout. 95% confidence intervals for the C-index were estimated by 1,000-replicate bootstrap resampling of the validation set. Model calibration was assessed with the integrated calibration index and the Brier score at 24 months, before and after *post-hoc* Platt recalibration (21–23). Clinical utility was assessed by decision-curve analysis, comparing the net benefit of the day-100 chronic GVHD model with treat-all and treat-none strategies across a range of threshold probabilities. As a temporal sensitivity analysis (21–23), the model for chronic GVHD was additionally retrained on patients transplanted between 2010 and 2015 and validated on those transplanted between 2016 and 2018. For every endpoint, the models estimate the risk of the corresponding event within 24 months—for all-cause mortality, the probability of death from any cause—and model discrimination was quantified by the inverse-probability-of-censoring-weighted (IPCW) concordance index of Uno et al., truncated at 24 months (24). This prediction-model study is reported following the TRIPOD+AI reporting guideline (Transparent Reporting of a multivariable prediction model for Individual Prognosis Or Diagnosis–Artificial Intelligence), which provides harmonized reporting standards for clinical prediction models developed with regression or machine-learning methods; the present manuscript addresses its core domains, including the data source and participants, outcome and predictor definitions, missing-data handling, model development and internal validation, calibration, and model performance (25).

## Results

**Table 1** summarizes the baseline characteristics of the 14,430 patients; the complete variable set is provided in **Supplementary Table 1**. The median age of the patients at transplantation was 51 years (interquartile range (IQR): 39-60). The sources of stem cells were HLA-matched related bone marrow (BM) in 967 patients, HLA-matched related peripheral blood (PB) in 1,998 patients, haploidentical BM in 172 patients, haploidentical PB with PTCY in 320 patients, haploidentical PB without posttransplant cyclophosphamide (PTCY) in 778 patients, unrelated BM in 5,539 patients, unrelated PB in 366 patients, and cord blood in 4,237 patients; donor/graft source was missing in 53 cases. Myeloablative conditioning was administered in 9,390 patients and reduced-intensity conditioning in 5,040 patients. The most common indication of HSCT was intermediate-risk acute myeloid leukemia (AML) in complete remission (CR, n=3,233) followed by acute lymphoblastic leukemia in first CR (n=2,164) and intermediate-risk AML in non-CR (n=1,618). The median follow-up period of survivors was 2.2 years (IQR: 0.9-4.5). Of the total cohort, 2,798, 4,960, and 5,540 patients developed grade II–IV aGVHD at days 30, 60, and 100, respectively, and 683, 1,327, and 1,575 patients developed grade III–IV aGVHD at the corresponding time points (**Supplementary Table 2**). Compared with the full pretransplant cohort (n=21,840), the day-100 landmark cohort had similar age and time from diagnosis to transplantation (**Supplementary Table 7**).

**Table 1:** Baseline characteristics of the study cohort (N = 14,430).

Characteristic	N = 14,430
Age, y — median (IQR)	51 (39–60)
Time from diagnosis to transplant, d — median (IQR)	211 (147–434)
Donor/graft source, no. (%)	
Related	2,965 (21%)
Haploidentical	1,270 (9%)
Unrelated marrow	5,539 (38%)
Unrelated peripheral blood	366 (3%)
Cord blood	4,237 (29%)
HLA mismatch by locus, GvH direction, no. (%)	
None (8/8 matched)	5,414 (38%)
A	322 (2%)
B	95 (1%)
C	513 (4%)
DRB1	1,535 (11%)
2 alleles	1,143 (8%)
≥3 alleles	3,097 (21%)
Missing	2,311 (16%)
HLA allele mismatch, total of 8, no. (%)	
None (matched)	5,137 (36%)
1 allele	2,447 (17%)
≥2 alleles	4,535 (31%)
Missing	2,311 (16%)
Sex mismatch, no. (%)	6,503 (45%)
ABO matching, no. (%)	
Matched	6,947 (48%)
Minor mismatch	3,021 (21%)
Major mismatch	2,908 (20%)
Bidirectional mismatch	1,502 (10%)
Performance status ≥2, no. (%)	858 (6%)
Primary disease, no. (%)	
AML	6,159 (43%)
ALL	2,964 (21%)
MDS	1,948 (13%)
CML/MPN	791 (5%)
Lymphoid/plasma-cell/other	2,568 (18%)
Myeloablative conditioning, no. (%)	9,390 (65%)
Total body irradiation, no. (%)	9,935 (69%)
*In vivo* T-cell depletion, no. (%)	1,453 (10%)
HCT-CI ≥3, no. (%)	2,113 (15%)
CMV serostatus, no. (%)	
Recipient-positive	8,520 (59%)
Recipient-negative	1,935 (13%)
Missing	3,975 (28%)

IQR, interquartile range; HCT-CI, Hematopoietic Cell Transplantation-specific Comorbidity Index; GvH, graft-versus-host. HLA typing was unavailable for 2,311 patients (16%); all missing covariate values were imputed for model training (see Methods). The two HLA rows are distinct model inputs: locus-resolved graft-versus-host-direction mismatch and total allele mismatch (0–8). Donor/graft source, ABO matching, sex matching and performance status were missing in 53, 52, 513, and 6 patients, respectively; all percentages are of the full cohort. Percentages may not total 100% because of rounding.

Using only pretransplant factors, SEM achieved the highest C-index across the endpoints evaluated (cGVHD, NRM, and ACM), with modest differences from the strongest individual base learners and overlapping confidence intervals (**Table 2**, the C-index for cGVHD/NRM/ACM-SEM: 0.574/0.652/0.642, Cox-PH: 0.553/0.635/0.615, Random Survival Forest: 0.564/0.638/0.613, XGBoost: 0.556/0.633/0.627, and Dynamic-DeepHit: 0.508/0.607/0.564). In paired bootstrap comparisons using pretransplant factors, the SEM significantly exceeded every individual learner for NRM and ACM, including the strongest (NRM versus Random Survival Forest, difference in C-index +0.013, 95% CI + 0.003 to +0.024, p=0.010; ACM versus XGBoost, +0.015, +0.004 to +0.025, p=0.010); for cGVHD, the SEM significantly exceeded the weaker learners but was statistically indistinguishable from the strongest (versus Random Survival Forest, +0.010, −0.005 to +0.025, p=0.19) (**Supplementary Table 4**). Within the day-100 cohort, the C-index of SEM for cGVHD, NRM, and ACM increased as accrued aGVHD information was incorporated.

**Table 2:** C-index for each prediction model.

	cGVHD				NRM				ACM			
Estimator	pre	d30	d60	d100	pre	d30	d60	d100	pre	d30	d60	d100
Stacked Ensemble	0.574	0.581	0.602	0.606	0.652	0.655	0.688	0.696	0.642	0.649	0.656	0.664
Dynamic-DeepHit	0.508	0.536	0.552	0.565	0.607	0.640	0.641	0.666	0.564	0.567	0.598	0.596
XGBoost	0.556	0.560	0.583	0.582	0.633	0.637	0.676	0.686	0.627	0.635	0.655	0.658
Cox-PH	0.553	0.566	0.591	0.597	0.635	0.642	0.663	0.677	0.615	0.622	0.631	0.638
Random Survival Forest	0.564	0.569	0.598	0.603	0.638	0.652	0.677	0.687	0.613	0.621	0.628	0.629

Values are inverse-probability-of-censoring-weighted (Uno) C-indices at a 24-month horizon. 95% confidence intervals for the Stacked Ensemble Model and pairwise SEM-versus-learner comparisons (differences, 95% CIs, and p-values) are provided in **Supplementary Tables 3, 4**.

The C-index (**Table 2**) for cGVHD/NRM/ACM: at day 30-0.581/0.655/0.649, at day 60-0.602/0.688/0.656, at day 100-0.606/0.696/0.664. For cGVHD, the C-index rose from 0.574 [0.555–0.593] using pretransplant variables alone to 0.606 [0.588–0.624] with day-100 aGVHD variables (+0.032); non-relapse mortality (0.652 to 0.696) and all-cause mortality (0.642 to 0.664) followed the same within-cohort pattern, confirming that the gain reflects the added posttransplant information rather than the conditioned cohort (**Supplementary Table 3**). Model calibration, assessed by the integrated calibration index (ICI) and the Brier score at 24 months, was modest before recalibration (ICI 0.07–0.11 across endpoints and landmarks) and improved substantially after a single *post-hoc* Platt recalibration step (**Supplementary Table 3**). In decision-curve analysis, the net benefit of the day-100 chronic GVHD model was similar to a treat-all strategy at low threshold probabilities and did not exceed it at higher thresholds (**Supplementary Table 6**), consistent with the modest discrimination described above. Bootstrap 95% confidence intervals for the Stacked Ensemble Model (validation set) ranged from 0.555 to 0.593 for cGVHD pretransplant to 0.672–0.723 for non-relapse mortality at day 100 (full intervals, **Supplementary Table 3**). In a temporal sensitivity analysis (training on 2010–2015 transplants and validating on 2016–2018 transplants), the landmark-improvement pattern was preserved: the cGVHD C-index rose from 0.566 [0.550–0.581] pretransplant to 0.594 [0.579–0.609] at day 100, with the same upward trend for NRM (0.658 to 0.707) and ACM (0.651 to 0.681), supporting temporal robustness.

**Figure 2:**
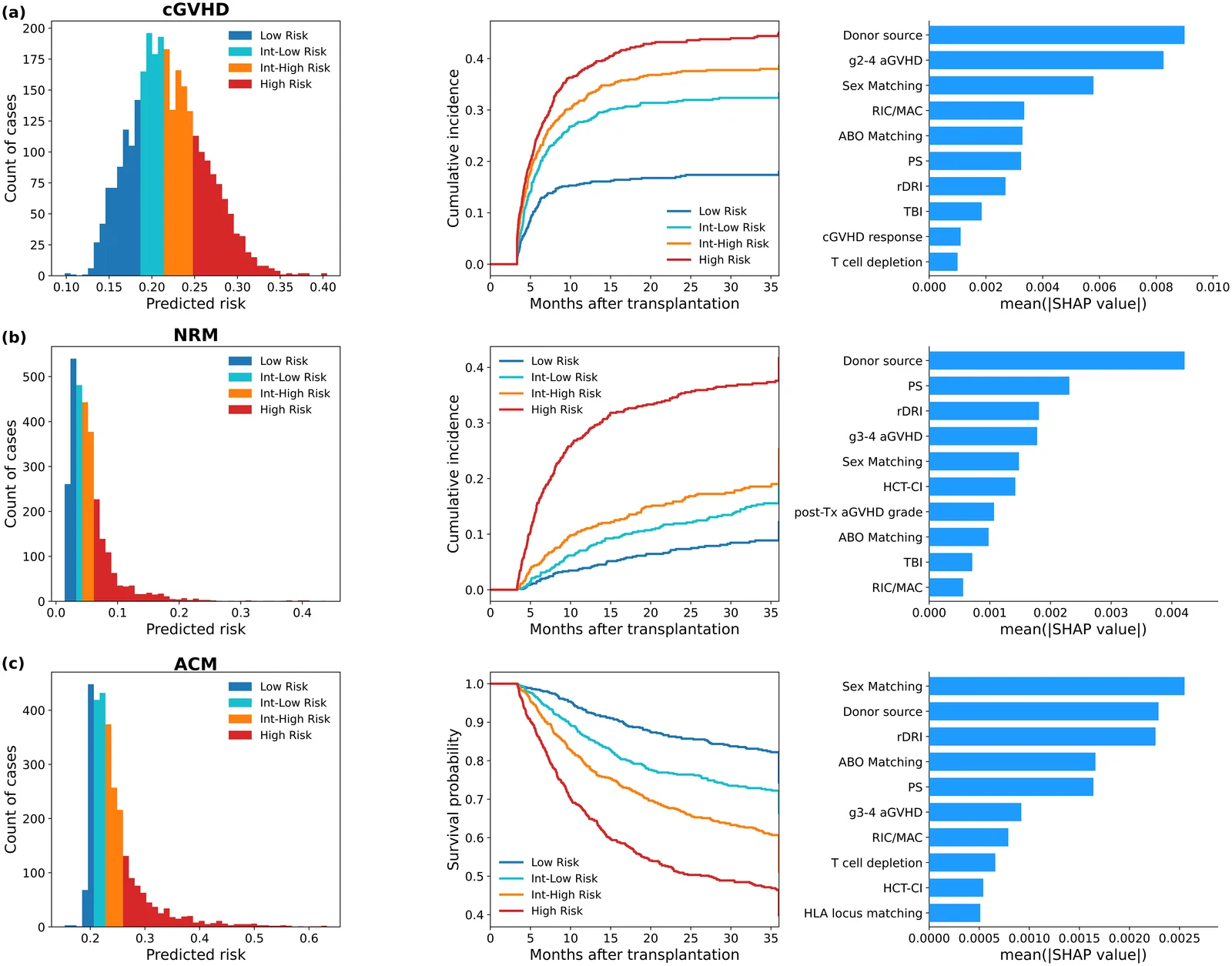
Predicted risk distributions, outcome curves, and mean SHAP values for cGVHD, NRM, and all-cause mortality. For cGVHD **(a)**, NRM **(b)**, and all-cause mortality **(c)**, predicted risk distributions (left), outcome curves (center), and mean SHAP values of the top 10 contributing variables (right) are shown for the validation cohort. Curves are displayed in the conventional direction for each endpoint: cumulative incidence for cGVHD and NRM, and the Kaplan–Meier overall survival function (1 − all-cause mortality) in panel **(c)**. The model was developed using pretransplant and day 100 posttransplant variables.

We stratified patients based on the prediction scores calculated by Stacked-Ensemble Model. Cumulative incidence and Kaplan–Meier curves from day 100 after transplantation showed clear separation of the four risk groups (**Figure 2**). Observed 24-month cGVHD incidence rose monotonically across SEM risk quartiles (16.5%, 30.2%, 35.9%, and 41.6%; **Supplementary Table 5**). SHapley Additive Explanations (SHAP) showed contribution of variables for predicting specific outcomes. Grade III to IV aGVHD and stage of aGVHD after treatment were important predictors of non-relapse mortality. Particularly, grade III to IV aGVHD were strongly associated with model predictions for ACM in line with pretransplant risk factors including sex matching, donor source, and disease/status groups. Grade II to IV aGVHD and treatment response of first-line therapy for aGVHD were important predictors of cGVHD.

## Discussion

Our study underscores the critical role of acute GVHD (aGVHD) events in shaping mid- to long-term posttransplantation outcomes. By employing SEM alongside classical statistical and machine learning approaches, we demonstrate that incorporating time-dependent posttransplant events significantly enhances predictive accuracy. These findings validate the importance of early-phase events in outcome prediction.

Consistent with previous reports, aGVHD strongly influences the development of chronic GVHD (cGVHD) and overall prognosis beyond day 100 posttransplantation (3). SHAP revealed that the most influential predictors vary by outcome. Grade III–IV aGVHD was a major determinant of non-relapse mortality and all-cause mortality, alongside pretransplant risk factors such as sex mismatch, donor source, and disease status. Additionally, both grade II–IV aGVHD and the response to first-line aGVHD therapy were associated with cGVHD development, aligning with prior findings (26).

While the inclusion of posttransplant events improved model performance, predictive accuracy remains limited. For cGVHD, emerging biomarkers reflecting early immunological shifts, such as changes in Th1/Th2/regulatory T cells, B cells, monocytes, and plasmacytoid dendritic cells, through cis- or trans-cell-to-cell interaction via chemokines or cytokines, may offer further predictive value. Early detection of these immune interactions could facilitate timely intervention and improve outcomes (8). Prior machine-learning approaches to transplant-outcome prediction include random survival forests, which outperformed Cox-based and registry risk scores for survival and non-relapse mortality in a myelofibrosis cohort and in a non-malignant inborn-errors cohort, and competing-risks survival trees applied to cytomegalovirus infection and non-relapse mortality (27–29). Building on the stacked-ensemble framework established in our prior work, the present study incorporates dynamic posttransplant acute-GVHD information across successive landmarks under a common competing-risk formulation.

Several limitations should be acknowledged explicitly. First, the analysis draws on a single national registry of Japanese transplant recipients with relatively homogeneous ancestry and standardized clinical practice; generalization to other populations and to centers with different supportive-care strategies will require external validation. Second, several posttransplant covariates (aGVHD stage before and after treatment, treatment response) were missing in a substantial fraction of cases and were handled by single imputation, which can attenuate effect sizes and partly explains the moderate gain from posttransplant variables for cGVHD. Third, the present model does not incorporate cellular or soluble immunological readouts, which remain the most plausible source of further predictive lift. Bootstrap and temporal split support robustness of the stacked ensemble model although not substituting for prospective external validation. Finally, SHAP values quantify each variable’s predictive contribution within the ensemble and do not establish causal effects on chronic GVHD or competing-event biology where our analysis showed the improvement of predictive accuracy by adding time-varying aGVHD-associated variables. For more explorative biomarker investigation, we might need to apply SEM for causal inference methods (30).

This study suggests the importance of early detection of inflammatory signals after HSCT. A natural next step is to integrate cellular and soluble biomarkers into the SEM framework. Candidate inputs include peripheral CXCL10 and plasmacytoid dendritic cell counts (8), regulatory T-cell reconstitution dynamics (6), and B-cell phenotypes capturing aberrant survival signals such as elevated BAFF (7). Embedding these immunological features alongside the clinical variables used here may convert today’s modest discrimination into clinically actionable risk thresholds for the very window, days 60–100, in which preemptive immunomodulation is most plausible. A complementary line of work has shown that posttransplant soluble biomarkers improve prediction of chronic GVHD and non-relapse mortality: the recent BIOPREVENT model combined seven day-90/100 plasma proteins—including CXCL9 and CXCL10—with baseline clinical variables in a competing-risks ensemble (31). That model relied on biomarkers from a single timepoint and, as its authors note, could not incorporate the evolving clinical course, including serial acute-GVHD severity, treatment, and response; the present study is complementary in exactly this dimension, capturing the dynamic acute-GVHD trajectory across days 30, 60, and 100. Integrating serial soluble biomarkers with dynamic acute-GVHD information within a single competing-risks ensemble is therefore a natural next step toward a dynamic, multilayer risk model for chronic GVHD and its downstream mortality.

In conclusion, we developed a novel SEM-based prediction model for mid-to-long-term outcomes following allogeneic HSCT, whose discrimination and calibration were at least comparable with traditional statistical models and other machine-learning algorithms. This highlights the prognostic significance of aGVHD and its treatment response in predicting cGVHD. Early identification and management of aGVHD are essential not only for immediate treatment but also for preventing downstream complications such as cGVHD, which are associated with poor long-term survival.

## Data Availability

The data are not publicly available because they exceed the scope of the recipient/donor consent for registry research use, and may be available from the corresponding author upon reasonable request with JSTCT/JDCHCT permission. Requests to access these datasets should be directed to jkanda16@kuhp.kyoto-u.ac.jp.
