# Supplementary material for: Development of a stacked ensemble model for risk stratification of chronic GVHD after allogeneic HSCT: Japanese nation-wide cohort study
Source: Front Immunol. 2026 Aug 25;17:1883360. doi: 10.3389/fimmu.2026.1883360 (PMC13549910; doi:10.3389/fimmu.2026.1883360)
Supplement: Supplementary file 1 [file Table1.docx]

**Supplementary Table 1 Pretransplant Patient Characteristics**

| **Category** |  | **Total** |
| --- | --- | --- |
|  |  | **N=14,430** |
| **Patient age, y (IQR)** |  | 51 (39-60) |
| **Time from diagnosis to transplantation, d (IQR)** |  | 211 (147-434) |
| **Donor/graft source, no. (%)** | **MRD-BM** | 967 ( 7%) |
|  | **MRD-PB** | 1,998 (14%) |
|  | **Haplo-BM** | 172 ( 1%) |
|  | **Haplo-PB with PTCY** | 320 ( 2%) |
|  | **Haplo-PB without PTCY** | 778 ( 5%) |
|  | **MUD-BM** | 3,063 (21%) |
|  | **PMUD-BM** | 1,969 (14%) |
|  | **MMUD-BM** | 507 ( 4%) |
|  | **MUD-PB** | 254 ( 2%) |
|  | **MMUD-PB** | 112 ( 1%) |
|  | **CB** | 4,237 (29%) |
|  | **Missing** | 53 ( 0%) |
| **Sex matching, no. (%)** | **Match** | 7,414 (51%) |
|  | **Mismatch** | 6,503 (45%) |
|  | **Missing** | 513 ( 4%) |
| **Performance Status, no. (%)** | **PS 0-1** | 13,566 (94%) |
|  | **PS 2-** | 858 ( 6%) |
|  | **Missing** | 6 ( 0%) |
| **ABO matching, no. (%)** | **Matched** | 6,947 (48%) |
|  | **Minor mismatch** | 3,021 (21%) |
|  | **Major mismatch** | 2,908 (20%) |
|  | **Major-Minor mismatch** | 1,502 (10%) |
|  | **Missing** | 52 ( 0%) |
| **Diagnosis and stage, no. (%)** | **AML intermediate-CR** | 3,233 (22%) |
|  | **AML intermediate-NCR** | 1,618 (11%) |
|  | **AML favorable-CR** | 614 ( 4%) |
|  | **AML favorable-NCR** | 207 ( 1%) |
|  | **AML adverse-CR** | 213 ( 1%) |
|  | **AML adverse-NCR** | 274 ( 2%) |
|  | **ALL CR1** | 2,164 (15%) |
|  | **ALL CR2-3** | 388 ( 3%) |
|  | **ALL NCR** | 412 ( 3%) |
|  | **MDS Low-Int-early** | 424 ( 3%) |
|  | **MDS Low-Int-advanced** | 101 ( 1%) |
|  | **MDS Low-Adv-early** | 163 ( 1%) |
|  | **MDS Low-Adv-advanced** | 19 ( 0%) |
|  | **MDS High-Int-early** | 610 ( 4%) |
|  | **MDS High-Int-advanced** | 216 ( 1%) |
|  | **MDS High-Adv-early** | 319 ( 2%) |
|  | **MDS High-Adv-advanced** | 96 ( 1%) |
|  | **CML CP** | 299 ( 2%) |
|  | **CML AP** | 24 ( 0%) |
|  | **CML BC** | 69 ( 0%) |
|  | **Ph-neg MPN** | 399 ( 3%) |
|  | **Indolent_B-NHL CR/PR** | 159 ( 1%) |
|  | **Indolent_B-NHL SD/PD** | 106 ( 1%) |
|  | **MCL CR** | 19 ( 0%) |
|  | **MCL PR** | 8 ( 0%) |
|  | **MCL SD/PD** | 15 ( 0%) |
|  | **Aggressive_B-NHL CR/PR** | 230 ( 2%) |
|  | **Aggressive_B-NHL SD/PD** | 136 ( 1%) |
|  | **T-NHL CR/PR** | 252 ( 2%) |
|  | **T-NHL SD/PD** | 176 ( 1%) |
|  | **BL CR** | 11 ( 0%) |
|  | **BL PR-PD** | 9 ( 0%) |
|  | **NK-NHL CR/PR** | 110 ( 1%) |
|  | **NK-NHL SD/PD** | 35 ( 0%) |
|  | **ATL CR/PR** | 435 ( 3%) |
|  | **ATL SD/PD** | 326 ( 2%) |
|  | **CLL CR/PR** | 9 ( 0%) |
|  | **CLL SD/PD** | 9 ( 0%) |
|  | **HL CR** | 22 ( 0%) |
|  | **HL PR** | 23 ( 0%) |
|  | **HL SD/PD** | 61 ( 0%) |
|  | **MM CR-PR** | 79 ( 1%) |
|  | **MM SD/PD** | 22 ( 0%) |
|  | **ALAL CR** | 240 ( 2%) |
|  | **ALAL NCR** | 76 ( 1%) |
| **Conditioning intensity, no. (%)** | **MAC** | 9,390 (65%) |
|  | **RIC** | 5,040 (35%) |
| **Total body irradiation, no. (%)** | **No** | 4,495 (31%) |
|  | **Yes** | 9,935 (69%) |
| **in vivo T cell depletion, no. (%)** | **No** | 12,977 (90%) |
|  | **Yes** | 1,453 (10%) |
| **HCT-CI, no. (%)** | **0** | 8,846 (61%) |
|  | **1** | 2,045 (14%) |
|  | **2** | 1,426 (10%) |
|  | **3** | 1,336 ( 9%) |
|  | **4** | 409 ( 3%) |
|  | **5** | 203 ( 1%) |
|  | **6** | 99 ( 1%) |
|  | **7** | 42 ( 0%) |
|  | **>=8** | 24 ( 0%) |
| **HLA locus matching, no. (%)** | **MUD88** | 5,414 (38%) |
|  | **A.mismatch** | 322 ( 2%) |
|  | **B.mismatch** | 95 ( 1%) |
|  | **C.mismatch** | 513 ( 4%) |
|  | **DRB1.mismatch** | 1,535 (11%) |
|  | **2 allele mismatch** | 1,143 ( 8%) |
|  | **7** | 3,097 (21%) |
|  | **Missing** | 2,311 (16%) |
| **HLA matching, no. (%)** | **0** | 5,137 (36%) |
|  | **1** | 2,447 (17%) |
|  | **2** | 1,076 ( 7%) |
|  | **3** | 1,593 (11%) |
|  | **4** | 1,360 ( 9%) |
|  | **5** | 361 ( 3%) |
|  | **6** | 121 ( 1%) |
|  | **7** | 22 ( 0%) |
|  | **8** | 2 ( 0%) |
|  | **Missing** | 2,311 (16%) |
| **CMV serostatus, no. (%)** | **R-D-** | 1,071 ( 7%) |
|  | **R+D-** | 3,659 (25%) |
|  | **R-D+** | 864 ( 6%) |
|  | **R+D+** | 4,861 (34%) |
|  | **Missing** | 3,975 (28%) |

**Supplementary Table 2. Posttransplant patient characteristics**

| **Category** |  | **Grade II to IV aGVHD, no. (%)** | | | | | |
| --- | --- | --- | --- | --- | --- | --- | --- |
|  |  | **Day 30** | | **Day 60** | | **Day 100** | |
|  |  | **No** | **Yes** | **No** | **Yes** | **No** | **Yes** |
|  |  | **N=11,654** | **N=2,798** | **N=9,470** | **N=4,960** | **N=8,890** | **N=5,540** |
| **Grade III to IV aGVHD, no. (%)** | **No** | 11,654 (100%) | 2,115 (76%) | 9,470 (100%) | 3,633 (73%) | 8,890 (100%) | 3,965 (72%) |
|  | **Yes** | 0 ( 0%) | 683 (24%) | 0 ( 0%) | 1,327 (27%) | 0 ( 0%) | 1,575 (28%) |
| **Corticosteroid, no. (%)** | **No** | 11,425 (98%) | 1,762 (63%) | 9,098 (96%) | 2,869 (58%) | 8,492 (96%) | 3,123 (56%) |
|  | **Yes** | 229 ( 2%) | 1,036 (37%) | 372 ( 4%) | 2,091 (42%) | 398 ( 4%) | 2,417 (44%) |
| **Anti-thymocyte globulin, no. (%)** | **No** | 11,650 (100%) | 2,766 (99%) | 9,464 (100%) | 4,856 (98%) | 8,885 (100%) | 5,393 (97%) |
|  | **Yes** | 4 ( 0%) | 32 ( 1%) | 6 ( 0%) | 104 ( 2%) | 5 ( 0%) | 147 ( 3%) |
| **Response, no. (%)** | **SD or PD** | 4 ( 0%) | 37 ( 1%) | 159 ( 2%) | 927 (19%) | 313 ( 4%) | 1,648 (30%) |
|  | **Improve** | 8 ( 0%) | 60 ( 2%) | 81 ( 1%) | 624 (13%) | 129 ( 1%) | 1,073 (19%) |
|  | **Missing** | 11,642 (100%) | 2,701 (97%) | 9,230 (97%) | 3,409 (69%) | 8,448 (95%) | 2,819 (51%) |
| **Pre-treatment aGVHD grade, y** | **0** | 1,698 (15%) | 236 ( 8%) | 404 ( 4%) | 96 ( 2%) | 50 ( 1%) | 24 ( 0%) |
|  | **1** | 241 ( 2%) | 61 ( 2%) | 408 ( 4%) | 144 ( 3%) | 452 ( 5%) | 174 ( 3%) |
|  | **2** | 23 ( 0%) | 864 (31%) | 18 ( 0%) | 1,780 (36%) | 10 ( 0%) | 2,041 (37%) |
|  | **3** | 2 ( 0%) | 279 (10%) | 1 ( 0%) | 523 (11%) | 1 ( 0%) | 615 (11%) |
|  | **4** | 2 ( 0%) | 26 ( 1%) | 0 ( 0%) | 55 ( 1%) | 0 ( 0%) | 62 ( 1%) |
|  | **Missing** | 9,688 (83%) | 1,332 (48%) | 8,639 (91%) | 2,362 (48%) | 8,377 (94%) | 2,624 (47%) |
| **Post-treatment aGVHD grade, y** | **0** | 823 ( 7%) | 560 (20%) | 239 ( 3%) | 420 ( 8%) | 39 ( 0%) | 88 ( 2%) |
|  | **1** | 7 ( 0%) | 2 ( 0%) | 68 ( 1%) | 226 ( 5%) | 108 ( 1%) | 415 ( 7%) |
|  | **2** | 0 ( 0%) | 32 ( 1%) | 1 ( 0%) | 291 ( 6%) | 2 ( 0%) | 495 ( 9%) |
|  | **3** | 0 ( 0%) | 47 ( 2%) | 1 ( 0%) | 191 ( 4%) | 0 ( 0%) | 277 ( 5%) |
|  | **4** | 0 ( 0%) | 4 ( 0%) | 1 ( 0%) | 36 ( 1%) | 1 ( 0%) | 49 ( 1%) |
|  | **Missing** | 10,824 (93%) | 2,153 (77%) | 9,160 (97%) | 3,796 (77%) | 8,740 (98%) | 4,216 (76%) |

**Supplementary Table 3. Stacked Ensemble Model discrimination and calibration.**

| **Endpoint** | **Landmark** | **C-index [95% CI]** | **ICI (pre -> Platt)** | **Brier 24 mo (pre -> Platt)** |
| --- | --- | --- | --- | --- |
| cGVHD | Pretransplant | 0.574 [0.555-0.593] | 0.091 -> 0.041 | 0.314 -> 0.279 |
| cGVHD | Day 30 | 0.581 [0.562-0.598] | 0.066 -> 0.035 | 0.297 -> 0.278 |
| cGVHD | Day 60 | 0.602 [0.584-0.621] | 0.099 -> 0.035 | 0.315 -> 0.273 |
| cGVHD | Day 100 | 0.606 [0.588-0.624] | 0.099 -> 0.037 | 0.315 -> 0.272 |
| NRM | Pretransplant | 0.652 [0.628-0.677] | 0.075 -> 0.032 | 0.209 -> 0.180 |
| NRM | Day 30 | 0.655 [0.632-0.682] | 0.105 -> 0.025 | 0.216 -> 0.179 |
| NRM | Day 60 | 0.688 [0.665-0.715] | 0.106 -> 0.041 | 0.213 -> 0.172 |
| NRM | Day 100 | 0.696 [0.672-0.723] | 0.112 -> 0.039 | 0.214 -> 0.170 |
| ACM | Pretransplant | 0.642 [0.622-0.660] | 0.086 -> 0.045 | 0.225 -> 0.221 |
| ACM | Day 30 | 0.649 [0.628-0.667] | 0.099 -> 0.038 | 0.246 -> 0.218 |
| ACM | Day 60 | 0.656 [0.637-0.675] | 0.087 -> 0.058 | 0.233 -> 0.215 |
| ACM | Day 100 | 0.664 [0.645-0.683] | 0.083 -> 0.052 | 0.231 -> 0.214 |
| cGVHD | Pretransplant (temporal) | 0.566 [0.550-0.581] | 0.023 -> 0.020 | 0.346 -> 0.327 |
| cGVHD | Day 100 (temporal) | 0.594 [0.579-0.609] | 0.033 -> 0.027 | 0.345 -> 0.322 |
| NRM | Pretransplant (temporal) | 0.658 [0.639-0.676] | 0.041 -> 0.026 | 0.256 -> 0.246 |
| NRM | Day 100 (temporal) | 0.707 [0.687-0.726] | 0.055 -> 0.038 | 0.266 -> 0.226 |
| ACM | Pretransplant (temporal) | 0.651 [0.636-0.667] | 0.066 -> 0.028 | 0.293 -> 0.269 |
| ACM | Day 100 (temporal) | 0.681 [0.665-0.697] | 0.080 -> 0.029 | 0.306 -> 0.260 |

*C-index, inverse-probability-of-censoring-weighted concordance index (Uno et al.) truncated at 24 months; 95% CI by 1,000-replicate bootstrap; ICI, integrated calibration index; Platt, post-hoc recalibration. Temporal: trained 2010-2015, validated 2016-2018.*

**Supplementary Table 4. Pairwise comparison of the Stacked Ensemble Model versus each individual learner.**

| **Endpoint** | **Model** | **Comparator** | **SEM** | **Learner** | **Δ C-index (SEM − learner) [95% CI]** | **p** |
| --- | --- | --- | --- | --- | --- | --- |
| cGVHD | Pretransplant | Cox-PH | 0.574 | 0.553 | +0.021 [+0.009, +0.034] | 0.002 |
|  |  | Random Survival Forest | 0.574 | 0.564 | +0.010 [-0.005, +0.025] | 0.194 |
|  |  | XGBoost | 0.574 | 0.556 | +0.019 [+0.007, +0.031] | 0.002 |
|  |  | Dynamic-DeepHit | 0.574 | 0.508 | +0.067 [+0.042, +0.093] | <0.001 |
| cGVHD | Day 100 | Cox-PH | 0.606 | 0.597 | +0.009 [-0.001, +0.019] | 0.074 |
|  |  | Random Survival Forest | 0.606 | 0.603 | +0.003 [-0.006, +0.013] | 0.532 |
|  |  | XGBoost | 0.606 | 0.582 | +0.024 [+0.011, +0.037] | <0.001 |
|  |  | Dynamic-DeepHit | 0.606 | 0.565 | +0.041 [+0.027, +0.056] | <0.001 |
| excGVHD | Pretransplant | Cox-PH | 0.604 | 0.582 | +0.022 [+0.009, +0.036] | <0.001 |
|  |  | Random Survival Forest | 0.604 | 0.577 | +0.027 [+0.009, +0.048] | 0.002 |
|  |  | XGBoost | 0.604 | 0.591 | +0.012 [-0.004, +0.030] | 0.136 |
|  |  | Dynamic-DeepHit | 0.604 | 0.553 | +0.051 [+0.025, +0.079] | 0.002 |
| excGVHD | Day 100 | Cox-PH | 0.646 | 0.626 | +0.020 [+0.010, +0.031] | <0.001 |
|  |  | Random Survival Forest | 0.646 | 0.624 | +0.021 [+0.008, +0.035] | 0.002 |
|  |  | XGBoost | 0.646 | 0.625 | +0.021 [+0.003, +0.038] | 0.022 |
|  |  | Dynamic-DeepHit | 0.646 | 0.612 | +0.034 [+0.017, +0.052] | <0.001 |
| NRM | Pretransplant | Cox-PH | 0.652 | 0.635 | +0.017 [+0.008, +0.027] | <0.001 |
|  |  | Random Survival Forest | 0.652 | 0.638 | +0.013 [+0.003, +0.024] | 0.010 |
|  |  | XGBoost | 0.652 | 0.633 | +0.019 [+0.002, +0.034] | 0.036 |
|  |  | Dynamic-DeepHit | 0.652 | 0.607 | +0.044 [+0.026, +0.064] | <0.001 |
| NRM | Day 100 | Cox-PH | 0.696 | 0.677 | +0.019 [+0.010, +0.027] | <0.001 |
|  |  | Random Survival Forest | 0.696 | 0.687 | +0.009 [-0.003, +0.022] | 0.150 |
|  |  | XGBoost | 0.696 | 0.686 | +0.010 [-0.001, +0.021] | 0.106 |
|  |  | Dynamic-DeepHit | 0.696 | 0.666 | +0.030 [+0.014, +0.047] | <0.001 |
| ACM | Pretransplant | Cox-PH | 0.642 | 0.615 | +0.027 [+0.013, +0.040] | 0.002 |
|  |  | Random Survival Forest | 0.642 | 0.613 | +0.029 [+0.018, +0.041] | <0.001 |
|  |  | XGBoost | 0.642 | 0.627 | +0.015 [+0.004, +0.025] | 0.010 |
|  |  | Dynamic-DeepHit | 0.642 | 0.564 | +0.077 [+0.059, +0.097] | <0.001 |
| ACM | Day 100 | Cox-PH | 0.664 | 0.638 | +0.026 [+0.015, +0.038] | <0.001 |
|  |  | Random Survival Forest | 0.664 | 0.629 | +0.035 [+0.023, +0.046] | <0.001 |
|  |  | XGBoost | 0.664 | 0.658 | +0.006 [-0.003, +0.016] | 0.196 |
|  |  | Dynamic-DeepHit | 0.664 | 0.596 | +0.068 [+0.052, +0.084] | <0.001 |

Positive differences favor the Stacked Ensemble Model. P values are two-sided and derive from 1,000-replicate paired bootstrap resampling of the validation set.

**Supplementary Table 5. Observed chronic GVHD incidence by SEM risk quartile in day-100 model in validation set.**

| **Risk quartile** | **n** | **Observed 24-mo cGVHD incidence** |
| --- | --- | --- |
| Q1 (low) | 722 | 16.5% |
| Q2 | 721 | 30.2% |
| Q3 | 721 | 35.9% |
| Q4 (high) | 722 | 41.6% |

*Quartiles of the day-100 SEM score in the validation set; monotonic gradient supports the four-group stratification (Figure 1).*

**Supplementary Table 6. Decision-curve analysis: net benefit, day-100 chronic GVHD model (validation set).**

| **Threshold probability** | **Net benefit (SEM)** | **Net benefit (treat-all)** | **Net benefit (treat-none)** |
| --- | --- | --- | --- |
| 0.10 | 0.443 | 0.443 | 0.000 |
| 0.12 | 0.431 | 0.431 | 0.000 |
| 0.14 | 0.417 | 0.417 | 0.000 |
| 0.16 | 0.401 | 0.403 | 0.000 |
| 0.18 | 0.381 | 0.389 | 0.000 |
| 0.20 | 0.301 | 0.374 | 0.000 |
| 0.22 | 0.209 | 0.358 | 0.000 |
| 0.24 | 0.111 | 0.341 | 0.000 |
| 0.26 | 0.031 | 0.323 | 0.000 |
| 0.28 | 0.021 | 0.304 | 0.000 |
| 0.30 | -0.001 | 0.284 | 0.000 |

*Net benefit of SEM exceeds treat-none across threshold probabilities ~0.10-0.28; exploratory, not a stand-alone decision tool.*

**Supplementary Table 7. Baseline comparison of the pretransplant and day-100 landmark cohorts.**

| **Characteristic** | **Pretransplant cohort (n=21,840)** | **Day-100 cohort (n=14,430)** |
| --- | --- | --- |
| Age, years — median (IQR) | 52 (40-61) | 51 (39-60) |
| Time diagnosis-to-transplant, days — median (IQR) | 215 (147-435) | 211 (147-434) |

*Across the 12 categorical pretransplant variables, the largest absolute difference in any single category proportion between cohorts was 4.2 percentage points, indicating broadly similar baseline composition; per-variable characteristics follow the structure and labels of Supplementary Table 1.*
